# Temporal trends of cervical cancer demographics: a CDC WONDER database study

**DOI:** 10.3389/fonc.2025.1567305

**Published:** 2025-07-18

**Authors:** Grace Folino, Isabella Zent, Lillian Eason, Vikram Murugan, Taylor Billion, Ali Bin Abdul Jabbar, Mohsin Mirza, Abubakar Tauseef

**Affiliations:** ^1^ Department of Internal Medicine, Creighton University School of Medicine, Omaha, NE, United States; ^2^ Internal Medicine Department, Creighton University School of Medicine, Omaha, NE, United States

**Keywords:** CDC WONDER, cervical cancer, HPV, demographics, health disparities

## Abstract

**Introduction:**

Despite advancements in cervical cancer screening and HPV vaccines, demographic disparities perpetuate the burden of cervical cancer. The aim of this study is to utilize the most up-to-date CDC WONDER data of cervical cancer mortality to provide a comprehensive temporal analysis of demographic variables and account for patients missed in other database studies. In doing so, temporal trends found in this study may be used to guide future efforts and studies to understand nuanced barriers to cervical cancer screening and prevention.

**Methods:**

With CDC WONDER Data, cervical cancer-related mortality was assessed in the U.S. from 1999 to 2023. Using age-adjusted mortality rates (AAMR), temporal trends were analyzed using the Joinpoint Regression Program for women 25 years and older across race, census regions, urban/rural residence, and states. Annual percentage change (APC) and average annual percentage change (AAPC) were calculated with 95% confidence intervals.

**Results:**

Cervical cancer-related mortality declined over the study period with an AAPC of –1.043*. Between 2015 and 2023, there was a concerning positive change in AAMR [APC of 0.1272 (95% CI –0.3393 to 1.7502)], though not statistically significant. Black or African American patients experienced the highest AAMR across races but maintained a decrease in mortality rate over the study period [AAPC of -2.670* (95% CI -2.931 to -2.356)]. Region and race analysis demonstrated Black or African American patients in the Northeast held the largest decline in AAMR [AAPC of –3.218* (95% CI –3.708 to –2.390)], while Hispanic or Latino and Black or African American patients in the South closely followed AAPC of –1.347* (–1.898 to –0.824) and –2.656* (95% CI –2.939 to -2.350), respectively]. Rural areas (NonCore and Micropolitan) and the Southern region displayed a concerning positive trend after 2009 and 2010, though not statistically significant [APC values of 0.772 (95% CI -0.328 to 4.888), 0.986 (95% CI –0.252 to 4.887), and 0.286 (95% CI –0.061 to 0.772), respectively].

**Conclusion:**

These findings underscore the need for targeted interventions with consideration of regional and racial temporal disparities in cervical cancer-related mortality.

## Introduction

Until recent innovation in screening and prevention, cervical cancer was one of the most common causes of cancer related deaths in women (1, 2). The development of the Pap smear significantly reduced the incidence of cervical cancer through early detection, with later improvements such as liquid based cytology and HPV DNA testing (3). Beginning in 2006, vaccination efforts against Human Papillomavirus (HPV) through the evolving Gardasil vaccines provided increasing strain coverage in the United States and offered another tool for prevention (4). Despite these advancements, demographic disparities continue to significantly influence the cervical cancer burden. For instance, minority populations, specifically Black patients, are shown to have a higher cervical cancer incidence and mortality (5). In addition, patients with lower socioeconomic status and residing in remote areas have worse outcomes due to varying vaccination and screening rates (6).

Current literature on cervical cancer demographics and prognostic factors focus on the analysis of the data derived from the Surveillance, Epidemiology, and End Results Database (SEER), the National Program for Cancer Registries (NPCR), the US Cancer Statistics Public Use Database, the Behavioral Risk Factor Surveillance System, the North American Association of Central Cancer Registries, and the Center for Disease Control and Prevention Wide ranging Online Data for Epidemiological Research (CDC WONDER) (4, 7–17). One of the most recent database studies used data from the NPCR and SEER from 2001 to 2014 to determine cervical cancer incidence before and after the introduction of HPV vaccination in US females aged from 15 to 34 years old. This age range excludes ages 35 to 44, the most frequent age range to be diagnosed with cervical cancer, and US females who may have not received the HPV vaccination (4). CDC WONDER has been used to compare non-Hispanic African American and White women, but this does not account for the diversity seen in the US population (5). Another study investigated age-adjusted mortality rates across gynecological cancer-related deaths from 1999 to 2020; however, this study did not stratify types of gynecological cancers and therefore does not provide age-adjusted mortality rates specific to cervical cancer (17).

This study aims to analyze the most recent cervical cancer data derived from the CDC WONDER database to evaluate temporal trends in mortality, which may be used to guide future, demographically targeted efforts against cervical cancer mortality. Demographic factors such as race, ethnicity, age groups, region, state, and urbanization will be included in this analysis to provide a more comprehensive understanding of underlying disparities in the cervical cancer population. Utilizing the CDC WONDER database from 1999 to 2023 will allow this study to capture patients excluded from previous studies.

## Materials and methods

CDC WONDER was used to identify malignant neoplasm of the cervix uteri related deaths within the United States from 1999 to 2023. Data within the CDC WONDER is derived from cancer registries based on healthcare provider oncology reports and updated annually (18). Using mortality data extracted from death certificate records and the International Classification of Diseases, 10th Revision (ICD-10) codes is consistent with current literature investigating nationwide mortality trends (19, 20). Malignant neoplasm of the cervix related mortality was identified using ICD10, Clinical Malignant codes (C53). This analysis includes age groups 25 years and older, as malignant neoplasm of the cervix in individuals younger than 25 provides insufficient data through CDC WONDER, and current guidelines for cervical cancer screening begin at age 21 (3). CDC WONDER excludes any data that represents fewer than ten persons (18). All other cervical cancer patient data was included in this study to ensure comprehensive analysis of demographic trends. The study was exempt from institutional review board approval because CDC WONDER contains anonymized, publicly available data.

CDC WONDER stratifies data by race/ethnicity (non-Hispanic White, non-Hispanic Black or African American, non-Hispanic American Indian/Alaskan Native, non-Hispanic Asian/Pacific Islander, and Hispanic or Latino), ten-year age intervals (25-34, 35-44, 45-54, 55-64, 65-74, 75-84, 85 and above), urban-rural classification (utilizing the National Center for Health Statistics Urban-Rural Classification Scheme), and census regions (Northwest, Midwest, South, and West) as defined by the Census Bureau (21, 22).

The total number of subjects was 114,751. Malignant neoplasm of the cervix uteri-related crude and age-adjusted mortality rates (AAMR) were calculated. AAMR was reported for overall trends and for demographic subgroups such as race/ethnicity, region, and urban-rural classification to allow comparisons across populations with differing age structures. However, when stratified by specific age groups, only crude mortality rates were analyzed, as the CDC WONDER platform does not support the calculation of age-adjusted rates within age-restricted subgroups. This limitation likely arises from the incompatibility of adjusting age-specific groups against a standard population. Despite this, crude rates remain valuable for highlighting raw mortality patterns and were calculated by dividing the number of cervical cancer related deaths by the United States female population. The control for AAMR to allow for comparison of data was standardized using the United States population in the year 2000 (23). The Joinpoint Regression program (Joinpoint version 4.9.0.0 available from the National cancer Institute, Bethesda, Maryland) was used to analyze temporal trends in mortality and identify statistically significant changes in mortality trends by fitting linear models that best describe the data. This program applies a log-linear model to detect statistically significant changes in trend over time, automatically selecting the number and locations of Joinpoints using the Monte Carlo Permutation method to optimize model/best fit. The final model reports annual percentage (APC) with 95% confidence interval (CIs) for each segment between a Joinpoint and showcases the AAMRs calculated using the Monte Carlo Permutation test. Average Annual Percent Change (AAPC) and 95% CIs were calculated via the Joinpoint Regression Program by taking the weighted average of APC values. AAPCs demonstrated the reported mortality trend over the entire time period analyzed. APC and AAPCs were determined to be significant by using a two tailed t test with statistical significance set at p ≤ 0.05 (represented by asterisks ‘*’ in tables and figures). This study followed suit of previous CDC WONDER studies to calculate the AAMR and form Joinpoint Regressions (24–26).

## Results

### Overall

**Figure 1:**
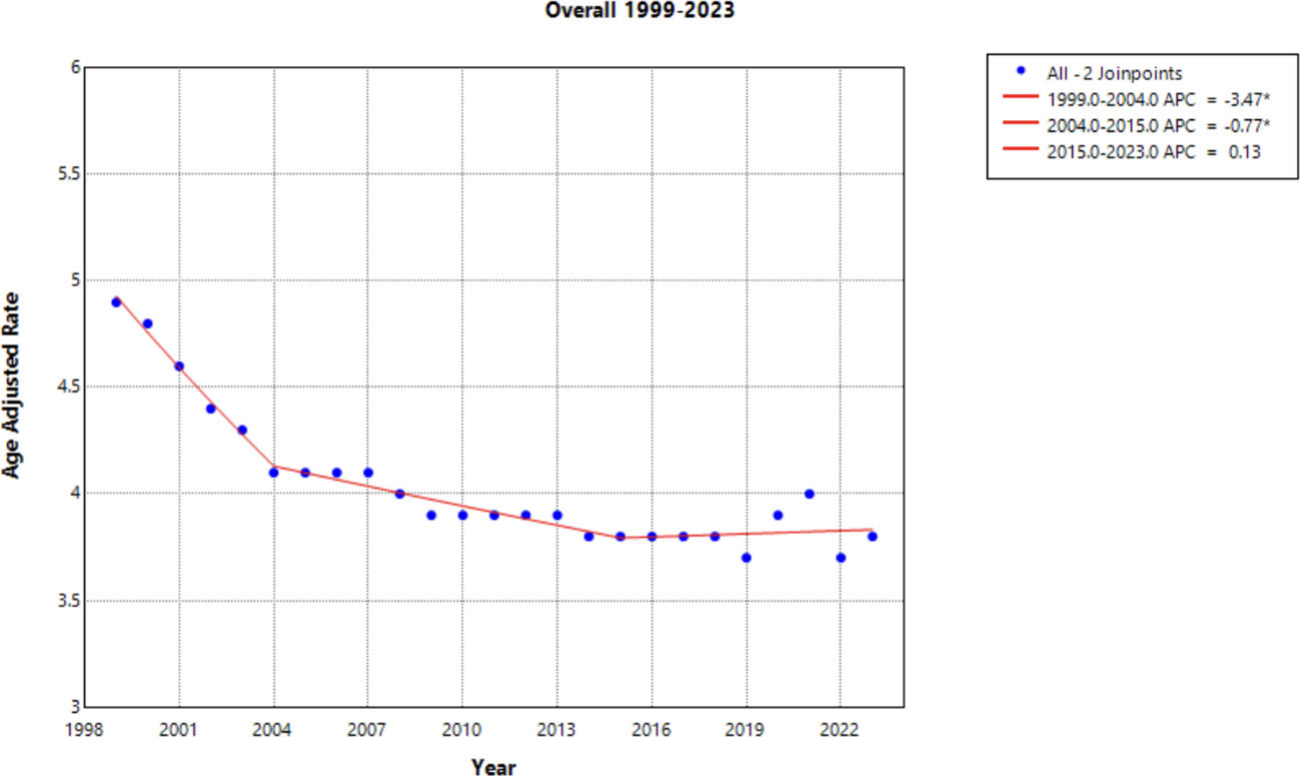
Joinpoint Model of Malignant Neoplasm of Cervix Uteri AAMR per 100,000 Residents, 1999-2023. Temporal trend segments are represented by APC values. (*APC Significant).

From 1999 to 2023, there were a total of 114,751 deaths from cervical cancer in the United States (**Figure 1**). Overall cervical cancer age-adjusted mortality rates (AAMR) declined significantly between 1999 and 2004 and again between 2004 and 2015 [APC of -3.472* (95% CI -4.808 to -2.690) and –0.771* (95% CI –1.975 to –0.467), respectively]. Between 2015 and 2023, there was no significant overall change in AAMR [APC of 0.127 (95% CI –0.339 to 1.750)] (**Supplementary Table 1**). Over the whole study timeframe, there was a significant average annual percent change (AAPC) of -1.043* (95% CI -1.179 to -0.897) (**Table 1**), demonstrating an overall consistent decreasing trend in age-adjusted mortality.

**Table 1:** Malignant neoplasms of cervix uteri AAPC values of investigated demographic factors.

Demographic factor	Cohort	AAPC (95% CI)
Overall	All (n=114,751)	-1.0432* (-1.1791 to -0.8967)
Region + All Races	Hispanic or Latino / Northeast (n=1954)	-2.6770* (-3.6439 to –1.6542)
	Hispanic or Latino / Midwest (n=881)	-2.2348* (-3.3196 to –0.9833)
	Hispanic or Latino / South (n=5381)	-1.3467* (-1.8983 to –0.8241)
	Hispanic or Latino / West (n=5485)	-1.8962* (-2.5844 to –1.2328)
	Asian or Pacific Islander / South (n=684)	-2.5352* (-3.392 to –1.5563)
	Asian or Pacific Islander / West (n=2085)	-2.0116* (-3.0037 to –0.8725)
	Black or African American / Northeast (n=3730)	-3.2178* (-3.7076 to –2.3896)
	Black or African American / Midwest (n=3740)	-2.4971* (-2.9977 to –2.0232)
	Black or African American / South (n=12515)	-2.6559* (-2.9388 to –2.3498)
	Black or African American / West (n=1460)	-2.2468* (-3.0637 to –1.4116)
	White / Northeast (n=12830)	-1.1832* (-1.8018 to –0.6446)
	White / Midwest (n=18616)	-0.7772* (-1.121 to –0.4289)
	White / South (n=29570)	0.1445 (-0.1427 to 0.44)
	White / West (n=13006)	-0.9655* (-1.4134 to –0.2204)
Urban/Rural	Large Central Metro (n=35445)	-1.7845* (-1.9727 to –1.5695)
	Large Fringe Metro (n=24091)	-1.1646* (-1.5079 to –0.784)
	Medium Metro (n=23491)	-0.7686* (-1.1804 to –0.4093)
	Small Metro (n=10853)	-0.7413* (-1.1046 to –0.3854)
	Micropolitan (Nonmetro) (n=11708)	-0.6708* (-1.1927 to –0.1695)
	NonCore (Nonmetro) (n=9163)	-0.7189* (-1.3136 to –0.1215)
Region	Northeast (n=19269)	-2.1749* (-2.5045 to –1.5992)
	Midwest (n=23908)	-1.0734* (-1.37 to –0.7959)
	South (n=48680)	-0.6836* (-0.8672 to –0.5076)
	West (n=22894)	-1.2239* (-1.5719 to –0.816)
Race	American Indian or Alaska Native (n=895)	-0.5921 (-1.8141 to 0.6796)
	Asian or Pacific Islander (n=4311)	-2.1138* (-2.9784 to –1.1858)
	Black or African American (n=21445)	-2.6700* (-2.9309 to –2.3562)
	White (n=74022)	-0.7234* (-0.8901 to –0.5819)
	Hispanic or Latino (n=13701)	-1.8053* (-2.1931 to –1.4169)
	Not Hispanic or Latino (n=100784)	-1.1622* (-1.479 to –0.9066)
Age Groups	25–34 years (n=5096)	-2.4812* (-3.5132 to –1.7191)
	35–44 years (n=15573)	-0.1934 (-0.566 to 0.1353)
	45–54 years (n=24170)	-0.7490* (-1.0569 to –0.294
	55–64 years (n=25065)	-1.1401* (-1.3439 to –0.9562)
	65–74 years (n=20569)	-1.1078* (-1.5241 to –0.6846)
	75–84 years (n=15393)	-1.4212* (-1.9463 to –0.9455)
	85+ years (n=8885)	-2.3788* (-2.8461 to –1.8217)

*Indicates significance at p ≤ 0.05. CI, Confidence Interval.

### Race

**Figure 2:**
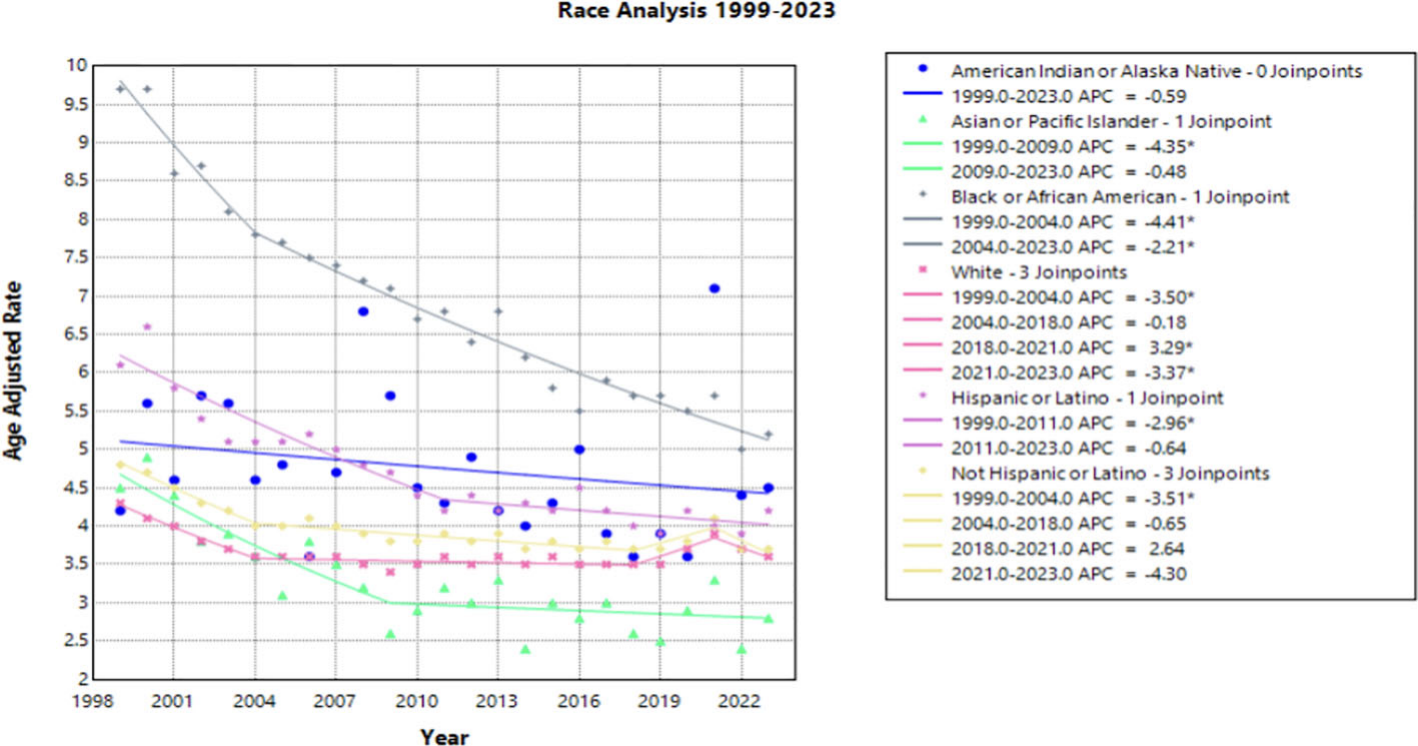
Joinpoint Model of Malignant Neoplasm of Cervix Uteri AAMR per 100,000 Residents by Race, 1999-2023. Temporal trend segments are represented by APC values. (*APC Significant).

When the data was stratified by race, the Black or African American population experienced the largest decline in AAMR across the study period, yet this same population consistently had the highest AAMR in comparison to other racial categories (**Figure 2**). This decline in AAMR is reflected in an AAPC of -2.670* (95% CI -2.931 to -2.356) (**Table 1**), which is the largest AAPC by race. Like the overall trend in data, the declining AAMR for the Black or African American population was steepest between 1999 and 2004 with an APC of -4.413* (95% CI -8.543 to -2.759) (**Figure 2**). This trend of significant decline continued between 2004 and 2023 with an APC of –2.206* (95% CI –2.486 to –1.297) (**Supplementary Table 1**). In contrast, the Asian or Pacific Islander population had many of the lowest AAMR values by race across this study period (**Figure 2**). This population experienced a significant decline in AAMR from 1999 to 2009 with an APC of -4.349* (95% CI -12.868 to -2.164); however, there was no significant decline following 2009 for the rest of the study period (**Figure 2**, **Supplementary Table 1**).

The White population experienced both significant declines and significant increases in AAMR over this time interval. Significant declines occurred between 1999 to 2004 and 2021 to 2023 [APC values of -3.498* (95% CI -4.703 to -2.696) and -3.370* (95% CI -0.5449 to -0.862), respectively]. However, from 2018 to 2021, this population experienced a significant increase in AAMR with an APC of 3.286* (95% CI 1.568 to 4.212) (**Figure 2**). The Hispanic or Latino population experienced a significant decline in AAMR between 1999 and 2011 with an APC of -2.957* (95% CI -5.339 to -2.170), followed by nonsignificant increases and decreases through 2023 [APC of –0.640 (95% CI –1.424 to 1.867] (**Figure 2**, **Supplementary Table 1**).

Interestingly, the only group by race that did not have an overall significant AAPC during the study period was the American Indian or Alaska Native population. In fact, there was no period during the timeframe of this study where the American Indian or Alaska Native population experienced a significant decline in AAMR (**Figure 2**).

### Race and region

**Figure 3:**
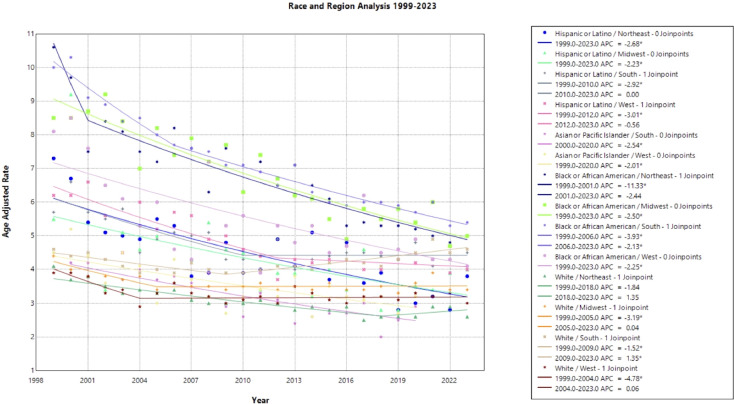
Joinpoint Model of Malignant Neoplasm of Cervix Uteri AAMR per 100,000 Residents by Region and Race, 1999-2023. Temporal trend segments are represented by APC values. (*APC Significant).

When stratifying race by region, each subset demonstrates a gradual decline in mortality rate over the time interval. Notably, Black and African American patients in the Northeast held the largest decline in AAMR, with an AAPC of –3.218* (95% CI –3.708 to –2.390) from 1999 to 2023. Hispanic or Latino patients in the Northeast and Black or African American patients in the South followed, with an AAPC of –2.677* (95% CI –3.644 to –1.654) and –2.656* (95% CI –2.939 to -2.350), respectively. All other race by region categories experienced a slight decline in mortality from 1999 to 2023, except for White women in the Midwest, South, and the West, in which the AAPC was less than one representing minimal to no change (**Figure 3**, **Table 1**). Black or African American patients in the South, Midwest, and Northeast maintained the highest AAMR across the period analyzed (**Figure 3**).

### Urban/Rural

**Figure 4:**
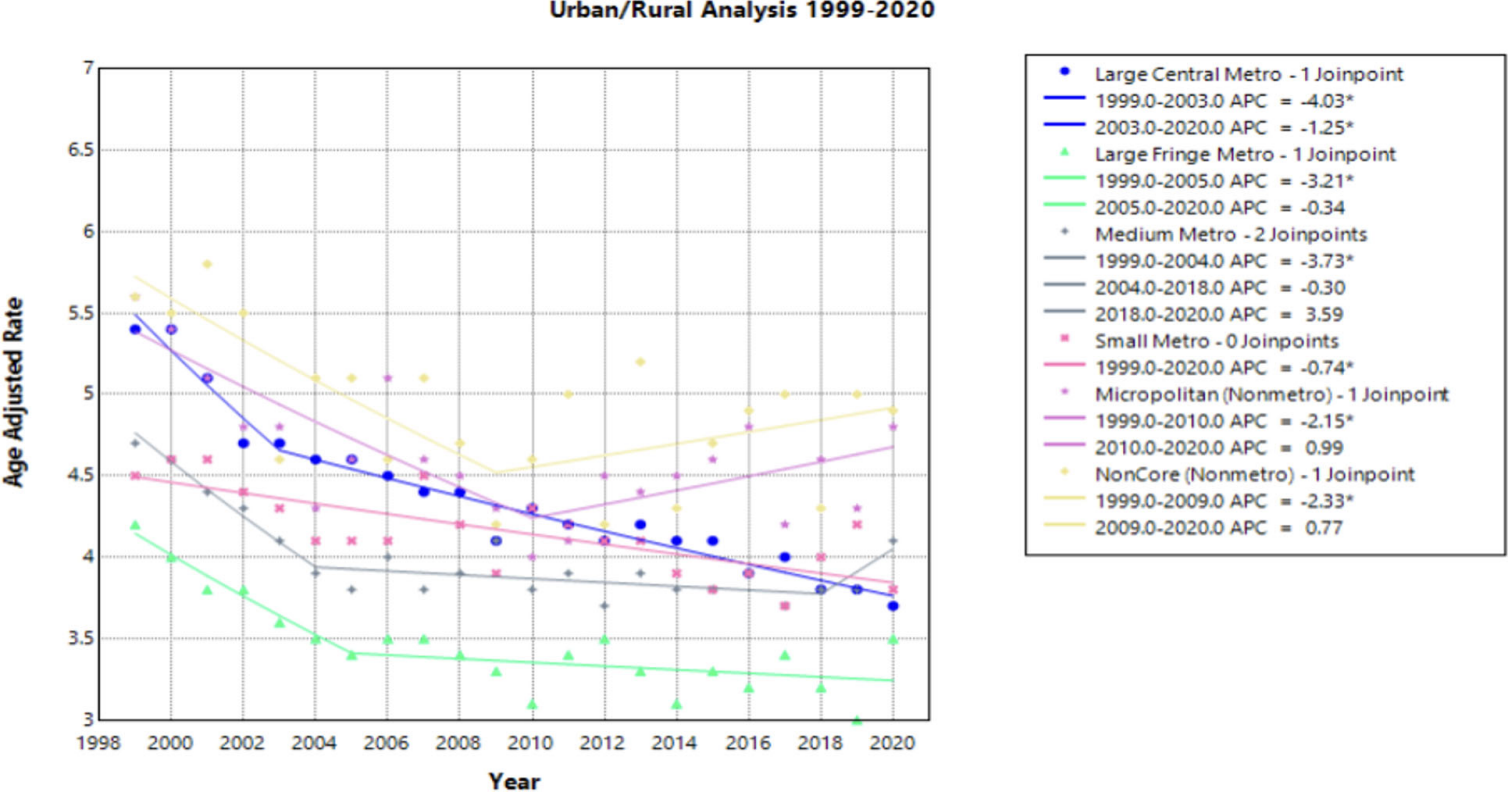
Joinpoint Model of Malignant Neoplasm of Cervix Uteri AAMR per 100,000 Residents by Urban or Rural, 1999-2023. Temporal trend segments are represented by APC values. (*APC Significant).

Of the Urban/Rural populations analyzed, most communities experienced a similar trend of a significant decline from 1999 to the 2000’s, ranging from 2003 to 2010, followed by a relative plateau until 2020 (**Figure 4**). Notably, NonCore (Nonmetro) and Micropolitan (Nonmetro) displayed a positive trend after 2009 and 2010, though not statistically significant [APC values of 0.772 (95% CI -0.328 to 4.888) and 0.986 (95% CI –0.252 to 4.887), respectively] (**Figure 4**, **Supplementary Table 1**). The Large Central Metro population demonstrates a rapid decline from 1999 to 2003, followed by smaller, but significant decline until 2020 (**Figure 4**). This area also showed the largest decline overall with an AAPC of –1.784* (95% CI –1.973 to –1.570) from 1999 to 2020 (**Table 1**). This analysis is limited to the time frame of 1999 to 2020 due to the availability of CDC WONDER data.

### Census regions

**Figure 5:**
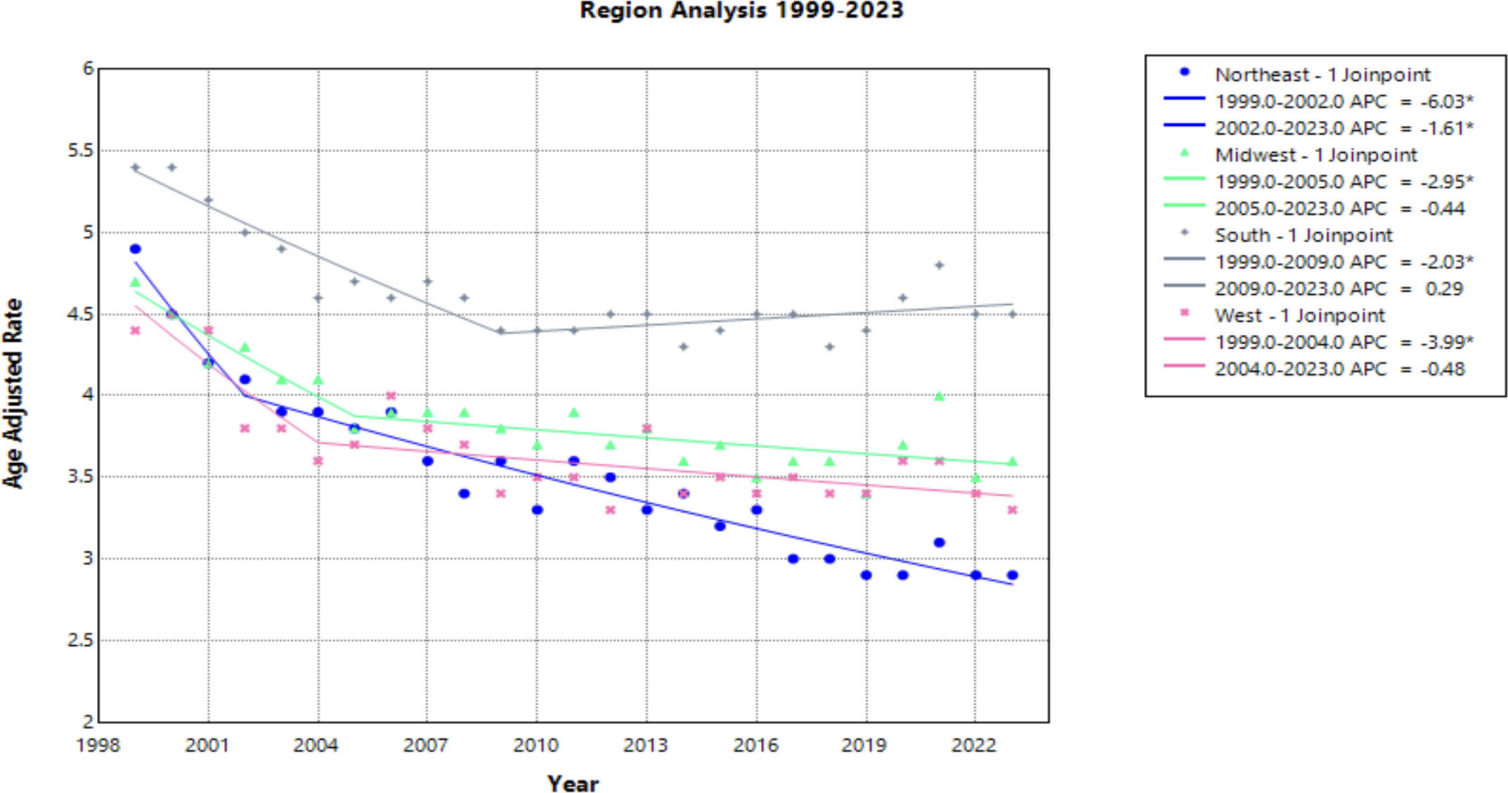
Joinpoint Model of Malignant Neoplasm of Cervix Uteri AAMR per 100,000 Residents by Census Regions, 1999-2023. Temporal trend segments are represented by APC values. (*APC Significant).

Each census region saw a decline in AAMR from 1999 to 2023, with the Northeast seeing the greatest decline [AAPC of –2.175* (95% CI –2.504 to –1.599)] (**Figure 5**, **Table 1**). This region was the only one to experience a continuous significant decline throughout the time interval analyzed. The South demonstrated a significantly negative trend in AAMR until 2009, when the APC became positive at a nonsignificant rate [APC of 0.286 (95% CI –0.061 to 0.772)]. This is the only region to experience a positive APC over the study period. All other regions have a slight decline of similar magnitude followed by a relative plateau until 2023 (**Figure 5**).

### Age groups

**Figure 6:**
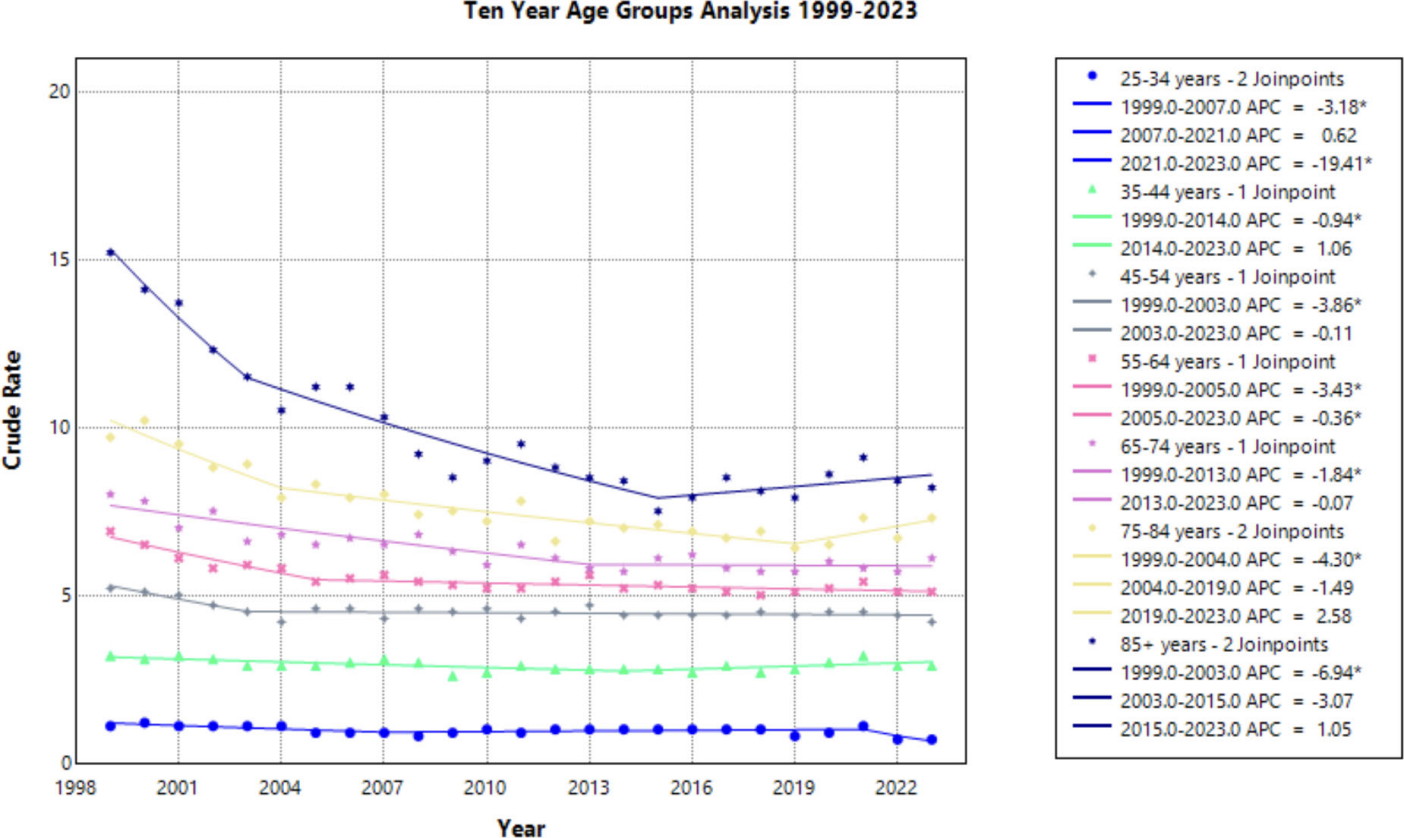
Joinpoint Model of Malignant Neoplasm of Cervix Uteri AAMR per 100,000 Residents by Age Group, 1999-2023. Temporal trend segments are represented by APC values. (*APC Significant).

Progressing age groups demonstrated higher crude mortality rate from 1999 to 2023 (**Figure 6**, **Table 1**). Crude mortality rate analysis was conducted on this data as Joinpoint Regression program prevents age adjusted mortality rate on the age group variable. The significance in AAPC values across this demographic variable may be attributed to lower prevalence of cervical cancer in younger age ranges, therefore skewing the statistical analysis. With understanding of low prevalence as a limitation in Joinpoint analysis, this result is not considered relevant to the aim of this study.

## Discussion

This study analyzed cervical cancer mortality data provided by the CDC WONDER database to determine temporal trends in demographic factors from 1999 to 2023. Overall average annual mortality rates of cervical cancer decreased from 1999 to 2015, with a notable change in the rate of decline from 1999 to 2004 (APC of –3.472; 95% CI –4.808 to –2.690) and 2004 to 2015 (APC of –0.771; 95% CI –1.975 to –0.467) (**Figure 1**, **Supplementary Table 1**). The sharp decline prior to 2004 may be due to the previous innovation in screening, as the development of the Pap smear and cytology allowed for detection of cervical cancer at earlier stages (27). The rate may have plateaued in recent years as the current screening tools may attained their efficacious value, indicating that other screening protocols may be needed to further advance early identification. Additionally, efforts to reduce the HPV burden through prophylactic vaccines demonstrated success in the cervical cancer population, but impact on mortality rates will likely be seen in the future (4, 28). Age groups of 25-34, 35-44, and 45–54 years old did not experience a notable decline in mortality (**Figure 6**). As established in previous literature, the median age for cervical cancer mortality is 60 years old (29). Therefore, it is less likely to see cervical cancer mortality impact these age groups.

Notable differences in cervical cancer mortality were found across racial populations. Black or African American individuals demonstrated significantly higher mortality than any other race each year, apart from 2021 (**Figure 2**). Previous studies indicate that Black or African American patients may have a higher rate of high-risk HPV positivity and advanced stage cervical cancer at the time of diagnosis, which may contribute to this trend (30–32). In recent years, studies have found that Black or African American patients attain equal or higher levels of cervical cancer screening, a trend that has shifted likely from targeted screening efforts of diverse communities (33–35). However, lower rates of follow up after abnormal Pap smears may contribute to the higher rate of mortality seen in this patient population (32, 33). Lack of follow up could be the consequence of structural, educational, or belief factors towards cervical cancer screening and detection. Previous studies demonstrate that African American, Hispanic, and publicly insured patients missed appointments more frequently than White and privately insured women (34, 36). Additionally, one study found that there was less knowledge among Black women of the characteristics of HPV and its role in cervical cancer (37). Establishing strong patient-provider communication may yield a path to providing education to this patient population. However, Black or African American women have a history of medical mistrust, which perpetuates barriers to receiving reproductive care and education (38, 39). Utilizing methods of culturally competent communication and creating a foundation of trust between patient and provider may assist in increasing education among Black and African American patients and deconstructing barriers to care (37).

In addition, a previous study found that providers who cared for larger proportions of Black patients were significantly more likely to practice in locations, such as community health centers, that were less likely to use liquid-based cytology, an innovation in cervical cancer screening that accounts for many Pap smears conducted in the United States. Other providers in this study who cared for a patient population of more than 25% Black patients and uninsured patients were significantly less likely to use HPV DNA testing (40, 41). Future efforts should be made to improve follow up protocol after an abnormal Pap smear across diverse patient populations to ensure equitable management of cervical cancer. Additionally, measures should be taken to equip providers managing diverse patient populations with adequate screening tools to fully utilize adequate diagnostic care (3).

Regionality and race were analyzed in a single Joinpoint to highlight structural barriers extending between race and region. Black or African American patients in the Northeast experienced the highest mortality in 1999, followed by Black or African American patients in the South after 2001 (**Figure 3**). Southern states previously reported less cervical cancer screening, which may be a result of lower socioeconomic status, limited healthcare access, and structural racism perpetuating both regional and racial cervical cancer disparities (6, 42). This finding is in accordance with previous studies investigating associations between race and region, where it was suggested that focused community interventions and targeted efforts in the older, Black patient population may reduce cervical cancer mortality (43, 44).

In contrast, the Asian or Pacific Islander communities experienced many of the lowest mortality rates across this timeframe, although demonstrated no significant decline (**Figure 2**). It should be acknowledged that the classification of Asian and Pacific Islander groups includes diverse subgroups; therefore, the CDC WONDER database may create a generalization of these statistics.

American Indian and Alaskan Native demonstrated a large amount of variance across this timeframe. This may be attributed to inconsistencies and misclassification on death certificates and could impact the data drawn from the CDC WONDER database (45). Training should be made available to providers in the future to correct these misclassifications, as recommended by the Department of Indigenous Health at the University of North Dakota (46).

NonCore (Nonmetro) and Micropolitan (Nonmetro) areas demonstrate a concerning trend towards increasing mortality after 2009 and 2010, respectively, though not statistically significant in this study (**Figure 4**). This is in accordance with recent literature, which demonstrated high incidence and mortality of cervical cancer in rural areas when compared to urban areas (47). Interestingly, the Southern region of the United States and rural areas (NonCore and Micropolitan) are the only demographic factors to demonstrate increasing mortality APC values after 2009 and 2010 (**Figures 4**, **5**). This could be attributed to socioeconomic factors impacting at-risk areas. One study reported an acceleration of rural hospital closures after 2010, likely from economic factors impacting profitability and staffing resources (48). The impact of rural hospital closure widens the gap to healthcare access in rural settings, perpetuating barriers to receiving both preventative screening and early management of cervical cancer (49–52). One study found that the regions most affected by these closures include rural communities in the East South Central and West South Central (53). Another study found that approximately 9% of women may experience a barrier to gynecologic malignancy care due to a geographic barrier (54). This is not an isolated issue in the United States, as studies have shown lower socioeconomic status and rural communities are less likely to receive a Pap smear and obtain established access to care (55). Distance to care and lack of access to specialty care services may negatively influence the prognosis of cervical cancer patients.

To address disparities in cervical cancer mortality, targeted efforts should be made to reduce barriers to care. Existing infrastructure in the South should be expanded upon to account for the persistently high mortality rate, with focused efforts among Black or African American patients to deconstruct the existing structural racism (56). Additionally, further initiatives to promote screening among underserved patients can reduce mortality through early identification. Studies demonstrate that programs such as HPV self-collection can increase cervical cancer screening among under screened patient populations (57–60). Furthermore, additional support for patients traveling to care centers and establishing gynecologic oncologist in rural settings may address the healthcare disparity experienced by rural patients (61–63). Existing healthcare centers should continue to focus efforts on building patient-provider relationships and forming strong communication to establish trust among all patient populations.

This study is limited by the reported data to CDC WONDER database, which may not fully represent the cervical cancer population and the individual factors impacting cervical cancer disparities. Additionally, this study examined all-cause mortality with malignant neoplasm of the cervix uteri rather than cervical cancer specific mortality. Analysis of age groups is limited to crude mortality by the Joinpoint regression program. Race and region analysis was limited by insufficient data provided by the CDC WONDER database.

## Conclusion

This study identifies temporal trends in cervical cancer across demographic factors from 1999 to 2023. The stark decrease in cervical cancer mortality has plateaued in recent years, which may indicate that previous innovation in screening has begun to reach its efficacious potential. Continued development of new screening techniques may address the slowing decline of mortality among the cervical cancer population. Variance of mortality rates demonstrates that the benefit experienced by the innovation in cervical cancer prevention and treatment is not equally shared across different populations. Black or African American patients, the Southern population, and rural communities are experiencing greater mortality, with a concerning turn towards an increasing mortality potentially as the result of barriers to care. These regional and racial disparities emphasize the crucial need for equitable screening and population-based research to guide protocols specific to diverse patient populations. The temporal trends highlighted in this study may be used as a guide to influence future studies and efforts in cervical cancer screening and prevention.

## Data Availability

Publicly available datasets were analyzed in this study. This data can be found here: https://wonder.cdc.gov/mcd.html.
