# Supplementary material for: Temporal trends of cervical cancer demographics: a CDC WONDER database study
Source: Front Oncol. 2025 Jul 18;15:1567305. doi: 10.3389/fonc.2025.1567305 (PMC12313508; doi:10.3389/fonc.2025.1567305)
Supplement: Supplementary file 1 [file Table1.docx]

| **Demographic Factor** | **Cohort** | **Lower Endpoint** | **Upper Endpoint** | **APC (95% CI)** | **P-Value** |
| --- | --- | --- | --- | --- | --- |
| Overall | All | 1999 | 2004 | -3.4717* (-4.8082, -2.6901) | < 0.000001 |
|  |  | 2004 | 2015 | -0.7713* (-1.9746, -0.4675) | 0.005199 |
|  |  | 2015 | 2023 | 0.1272 (-0.3393, 1.7502) | 0.538292 |
| Region + All Races | Hispanic or Latino / Northeast | 1999 | 2023 | -2.6770* (-3.6439, -1.6542) | < 0.000001 |
|  | Hispanic or Latino / Midwest | 1999 | 2023 | -2.2348* (-3.3196, -0.9833) | 0.0008 |
|  | Hispanic or Latino / South | 1999 | 2010 | -2.9166* (-7.0971, -1.7551) | < 0.000001 |
|  |  | 2010 | 2023 | 0.0014 (-0.8173, 1.9856) | 0.876625 |
|  | Hispanic or Latino / West | 1999 | 2012 | -3.0135* (-8.9343, -1.7918) | 0.028394 |
|  |  | 2012 | 2023 | -0.5592 (-2.0175, 6.2075) | 0.781044 |
|  | Asian or Pacific Islander / South | 2000 | 2020 | -2.5352* (-3.392, -1.5563) | < 0.000001 |
|  | Asian or Pacific Islander / West | 1999 | 2020 | -2.0116* (-3.0037, -0.8725) | 0.0012 |
|  | Black or African American / Northeast | 1999 | 2001 | -11.3283* (-16.6641, -2.6349) | 0.0004 |
|  |  | 2001 | 2023 | -2.4446 (-3.5822, 0.4787) | 0.056389 |
|  | Black or African American / Midwest | 1999 | 2023 | -2.4971* (-2.9977, -2.0232) | < 0.000001 |
|  | Black or African American / South | 1999 | 2006 | -3.9296* (-7.5496, -2.8245) | 0.0004 |
|  |  | 2006 | 2023 | -2.1265* (-2.4479, -0.6841) | 0.030394 |
|  | Black or African American / West | 1999 | 2023 | -2.2468* (-3.0637, -1.4116) | < 0.000001 |
|  | White / Northeast | 1999 | 2018 | -1.8396 (-3.9414, 4.2454) | 0.069986 |
|  |  | 2018 | 2023 | 1.3513 (-1.566, 8.0469) | 0.453509 |
|  | White / Midwest | 1999 | 2005 | -3.1882* (-7.3698, -1.4513) | < 0.000001 |
|  |  | 2005 | 2023 | 0.0398 (-0.3404, 0.8284) | 0.752649 |
|  | White / South | 1999 | 2009 | -1.5239* (-3.4781, -0.6837) | 0.0004 |
|  |  | 2009 | 2023 | 1.3534* (0.8225, 2.2799) | < 0.000001 |
|  | White / West | 1999 | 2004 | -4.7786* (-13.4903, -0.7376) | 0.008398 |
|  |  | 2004 | 2023 | 0.063 (-0.365, 1.3355) | 0.576685 |
| Urban/Rural | Large Central Metro | 1999 | 2003 | -4.0323* (-7.0744, -2.5459) | < 0.000001 |
|  |  | 2003 | 2020 | -1.2480* (-1.4789, -0.899) | 0.002 |
|  | Large Fringe Metro | 1999 | 2005 | -3.2065* (-7.063, -1.647) | < 0.000001 |
|  |  | 2005 | 2020 | -0.3359 (-0.7966, 0.7682) | 0.379524 |
|  | Medium Metro | 1999 | 2004 | -3.7305* (-7.0719, -1.4904) | 0.033193 |
|  |  | 2004 | 2018 | -0.3031 (-2.6646, 0.343) | 0.132374 |
|  |  | 2018 | 2020 | 3.5912 (-0.1665, 6.1823) | 0.095181 |
|  | Small Metro | 1999 | 2020 | -0.7413* (-1.1046, -0.3854) | 0.0004 |
|  | Micropolitan (Nonmetro) | 1999 | 2010 | -2.1537* (-5.5791, -1.1782) | 0.002 |
|  |  | 2010 | 2020 | 0.9865 (-0.2521, 4.8871) | 0.117976 |
|  | NonCore (Nonmetro) | 1999 | 2009 | -2.3332* (-6.7706, -1.1747) | 0.002 |
|  |  | 2009 | 2020 | 0.7717 (-0.3281, 4.8878) | 0.166367 |
| Region | Northeast | 1999 | 2002 | -6.0271* (-10.7863, -1.6889) | 0.013597 |
|  |  | 2002 | 2023 | -1.6119* (-2.3334, -0.3292) | 0.037592 |
|  | Midwest | 1999 | 2005 | -2.9534* (-6.3858, -1.5966) | < 0.000001 |
|  |  | 2005 | 2023 | -0.4386 (-0.7645, 0.1877) | 0.097181 |
|  | South | 1999 | 2009 | -2.0259* (-2.8395, -1.5189) | < 0.000001 |
|  |  | 2009 | 2023 | 0.2865 (-0.0611, 0.7723) | 0.104379 |
|  | West | 1999 | 2004 | -3.9924* (-8.8424, -1.6844) | 0.0004 |
|  |  | 2004 | 2023 | -0.4821 (-0.8648, 0.4805) | 0.145171 |
| Race | American Indian or Alaska Native | 1999 | 2023 | -0.5921 (-1.8141, 0.6796) | 0.358728 |
|  | Asian or Pacific Islander | 1999 | 2009 | -4.3494* (-12.8685, -2.1641) | 0.010398 |
|  |  | 2009 | 2023 | -0.485 (-1.6953, 5.0842) | 0.722655 |
|  | Black or African American | 1999 | 2004 | -4.4130* (-8.5433, -2.7589) | < 0.000001 |
|  |  | 2004 | 2023 | -2.2060* (-2.4856, -1.2971) | 0.013997 |
|  | White | 1999 | 2004 | -3.4977* (-4.7026, -2.6965) | < 0.000001 |
|  |  | 2004 | 2018 | -0.176 (-0.4777, 0.0669) | 0.129974 |
|  |  | 2018 | 2021 | 3.2856* (1.5678, 4.2125) | 0.015597 |
|  |  | 2021 | 2023 | -3.3696* (-5.4493, -0.8624) | 0.018396 |
|  | Hispanic or Latino | 1999 | 2011 | -2.9567* (-5.339, -2.1696) | 0.0008 |
|  |  | 2011 | 2023 | -0.6404 (-1.4236, 1.8666) | 0.309538 |
|  | Not Hispanic or Latino | 1999 | 2004 | -3.5144* (-6.3408, -2.2418) | 0.015997 |
|  |  | 2004 | 2018 | -0.6545 (-1.6507, 1.6355) | 0.117177 |
|  |  | 2018 | 2021 | 2.6385 (-2.2402, 3.9148) | 0.180364 |
|  |  | 2021 | 2023 | -4.2989 (-8.0094, 0.6808) | 0.154769 |
| Age Groups | 25-34 years | 1999 | 2007 | -3.1768* (-8.804, -1.2733) | 0.0008 |
|  |  | 2007 | 2021 | 0.6219 (-0.2062, 5.4733) | 0.131574 |
|  |  | 2021 | 2023 | -19.4067* (-29.2218, -6.2708) | 0.0004 |
|  | 35-44 years | 1999 | 2014 | -0.9408* (-2.6623, -0.4913) | 0.002799 |
|  |  | 2014 | 2023 | 1.0648 (-0.0022, 4.3582) | 0.05239 |
|  | 45-54 years | 1999 | 2003 | -3.8604* (-10.065, -0.7113) | 0.0004 |
|  |  | 2003 | 2023 | -0.1147 (-0.3184, 0.2853) | 0.481904 |
|  | 55-64 years | 1999 | 2005 | -3.4306* (-5.3289, -2.4328) | < 0.000001 |
|  |  | 2005 | 2023 | -0.3646* (-0.63, -0.0531) | 0.028794 |
|  | 65-74 years | 1999 | 2013 | -1.8432* (-5.488, -1.1224) | 0.023995 |
|  |  | 2013 | 2023 | -0.069 (-1.0697, 4.1122) | 0.976205 |
|  | 75-84 years | 1999 | 2004 | -4.2976* (-9.2327, -1.2727) | 0.045591 |
|  |  | 2004 | 2019 | -1.4939 (-4.7682, 0.8526) | 0.073185 |
|  |  | 2019 | 2023 | 2.5798 (-1.2331, 8.7184) | 0.186763 |
|  | 85+ years | 1999 | 2003 | -6.9369* (-12.7388, -3.233) | 0.024795 |
|  |  | 2003 | 2015 | -3.0702 (-6.7635, 4.5831) | 0.129974 |
|  |  | 2015 | 2023 | 1.0548 (-4.064, 7.1205) | 0.225955 |
