# Supplementary figures and images for: Temporal trends of cervical cancer demographics: a CDC WONDER database study
Source: Front Oncol. 2025 Jul 18;15:1567305. doi: 10.3389/fonc.2025.1567305 (PMC12313508; doi:10.3389/fonc.2025.1567305)

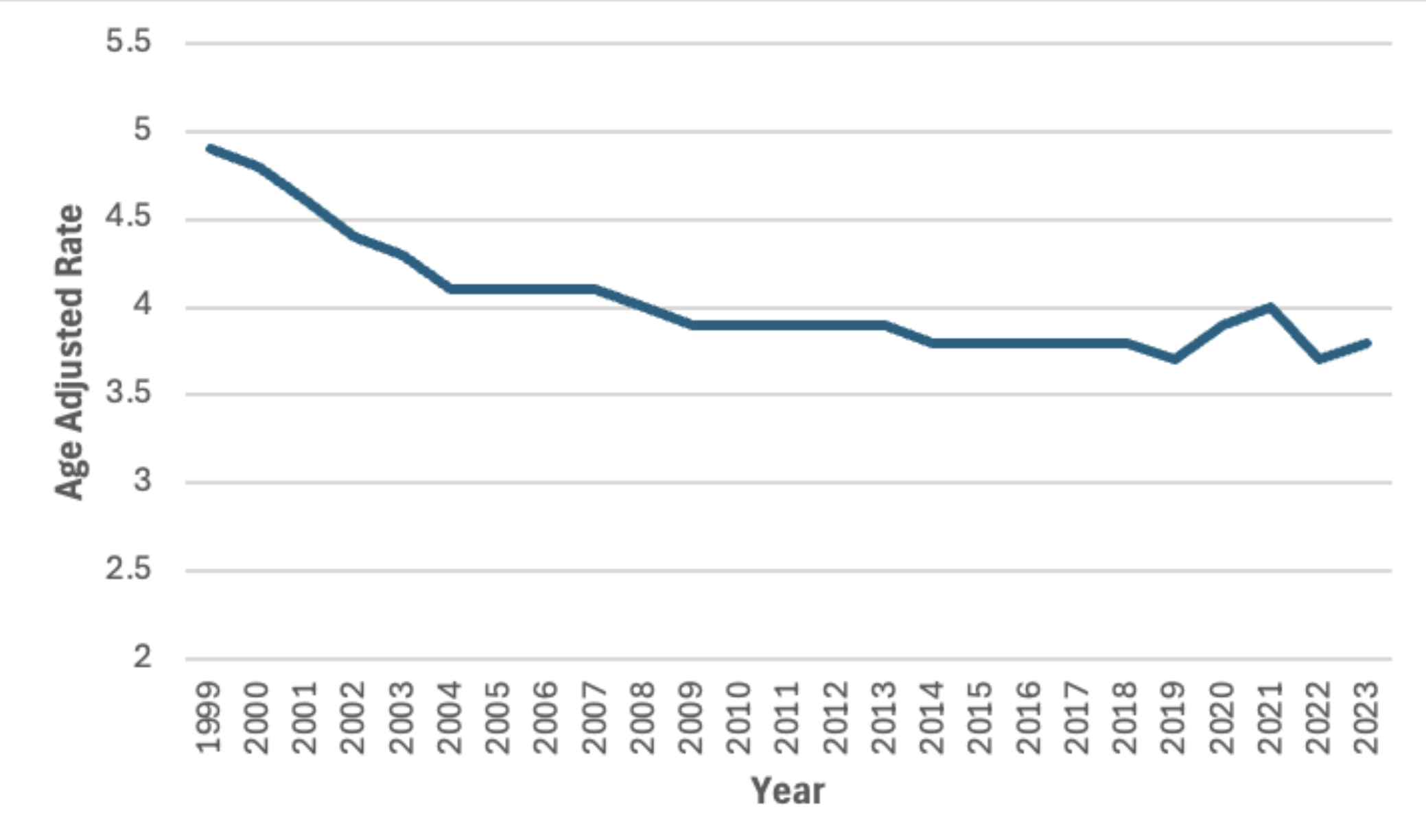

Supplement: Supplementary file 2 [file Image1.png]

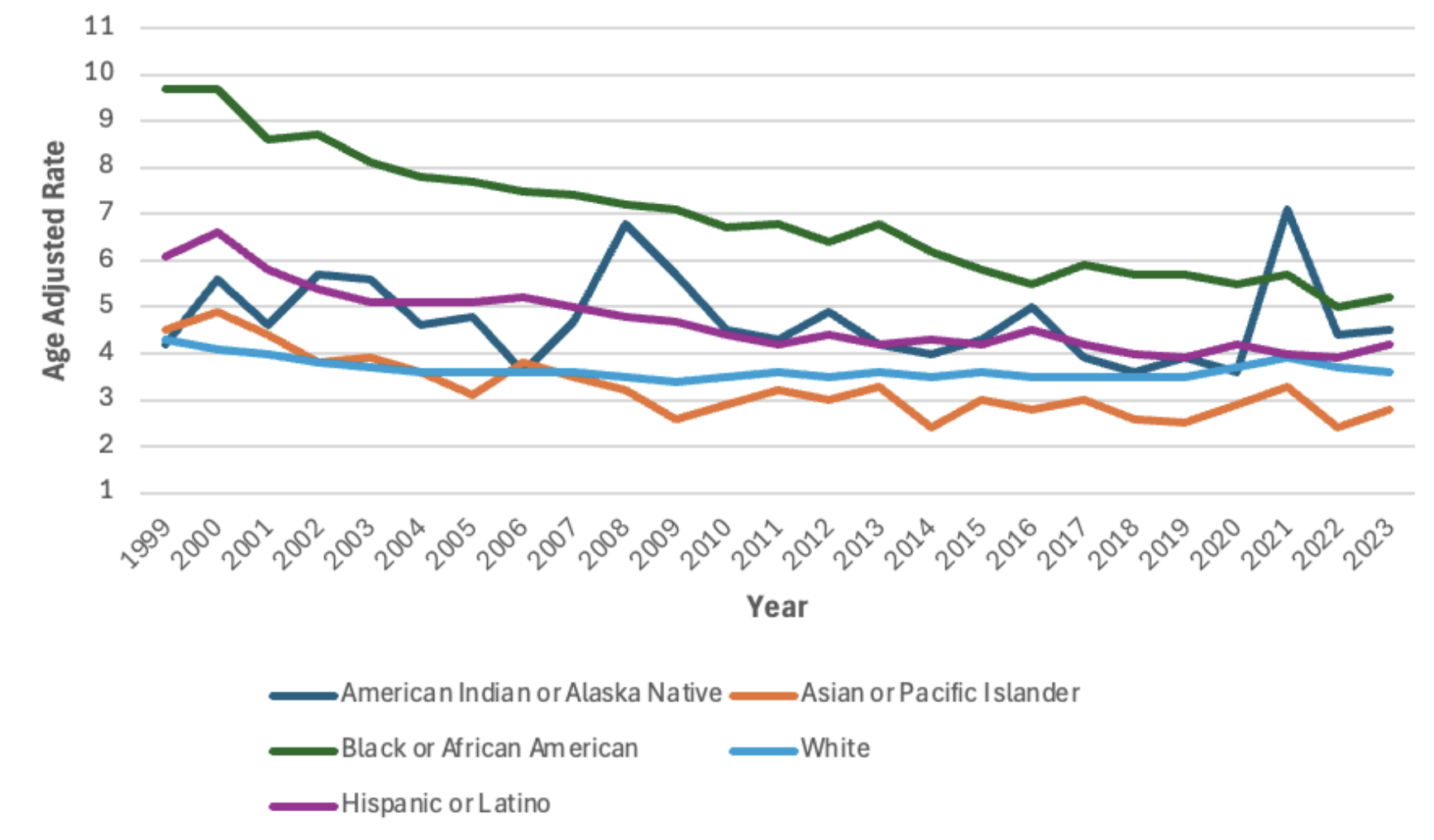

Supplement: Supplementary file 3 [file Image2.png]

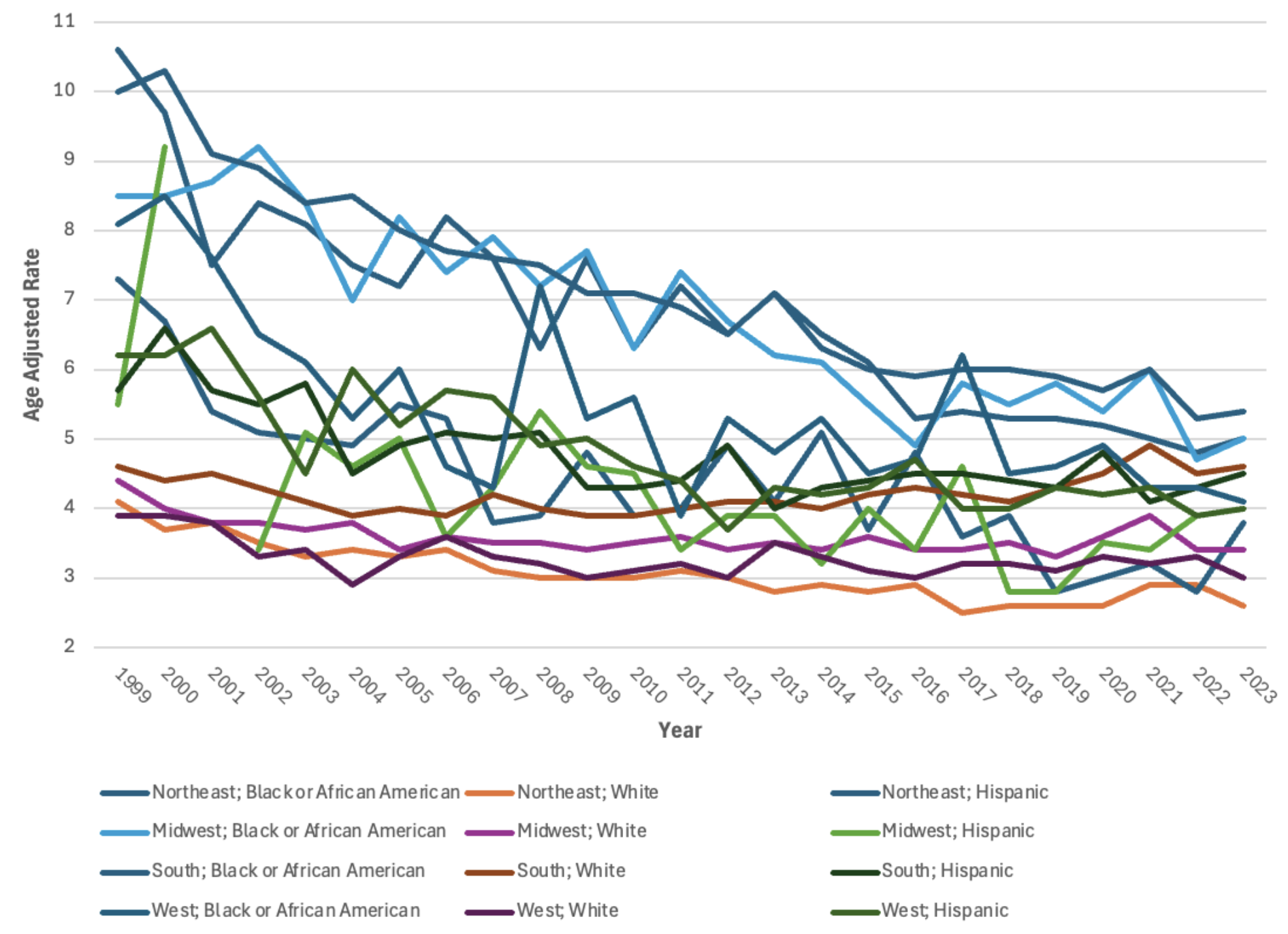

Supplement: Supplementary file 4 [file Image3.png]

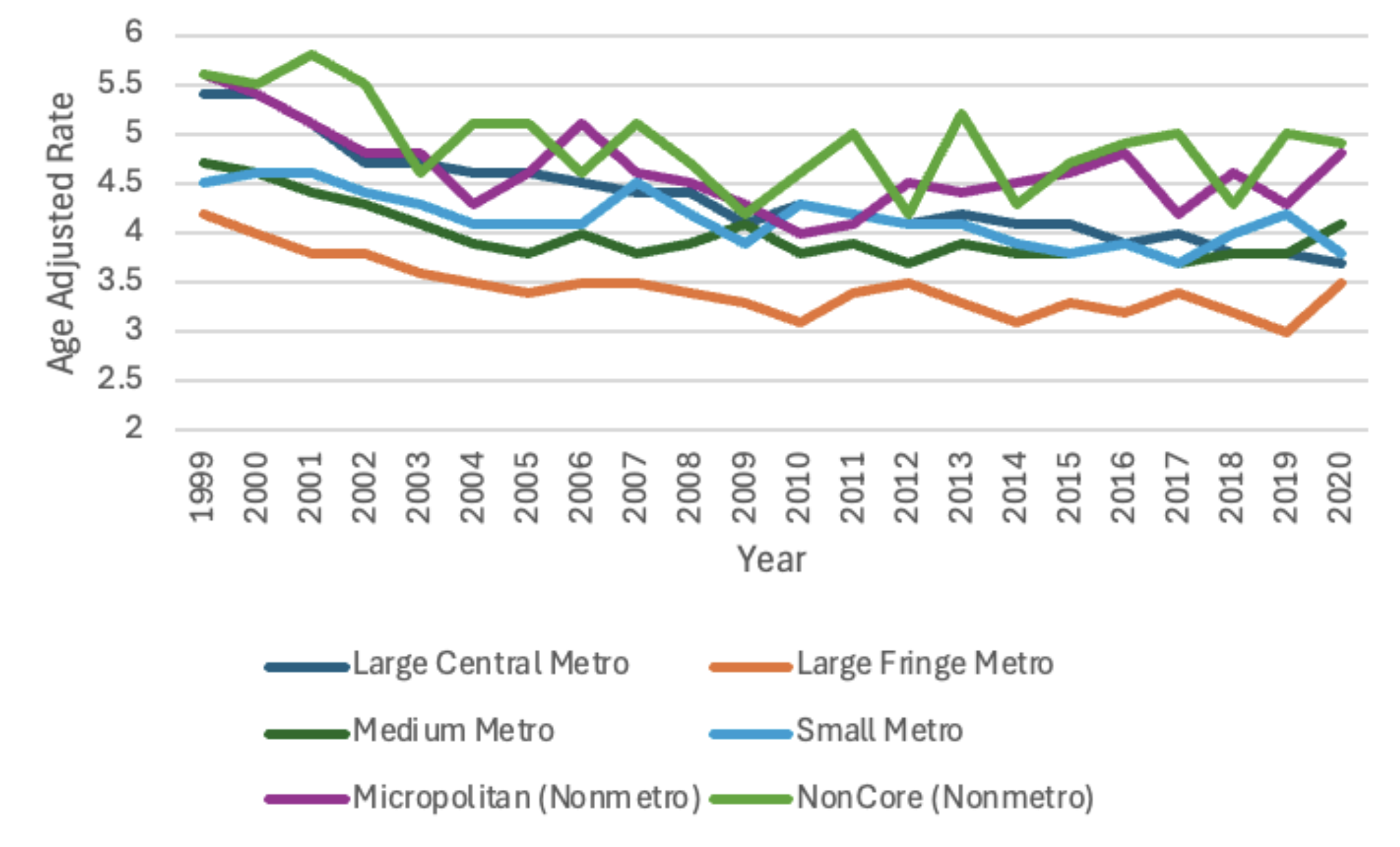

Supplement: Supplementary file 5 [file Image4.png]

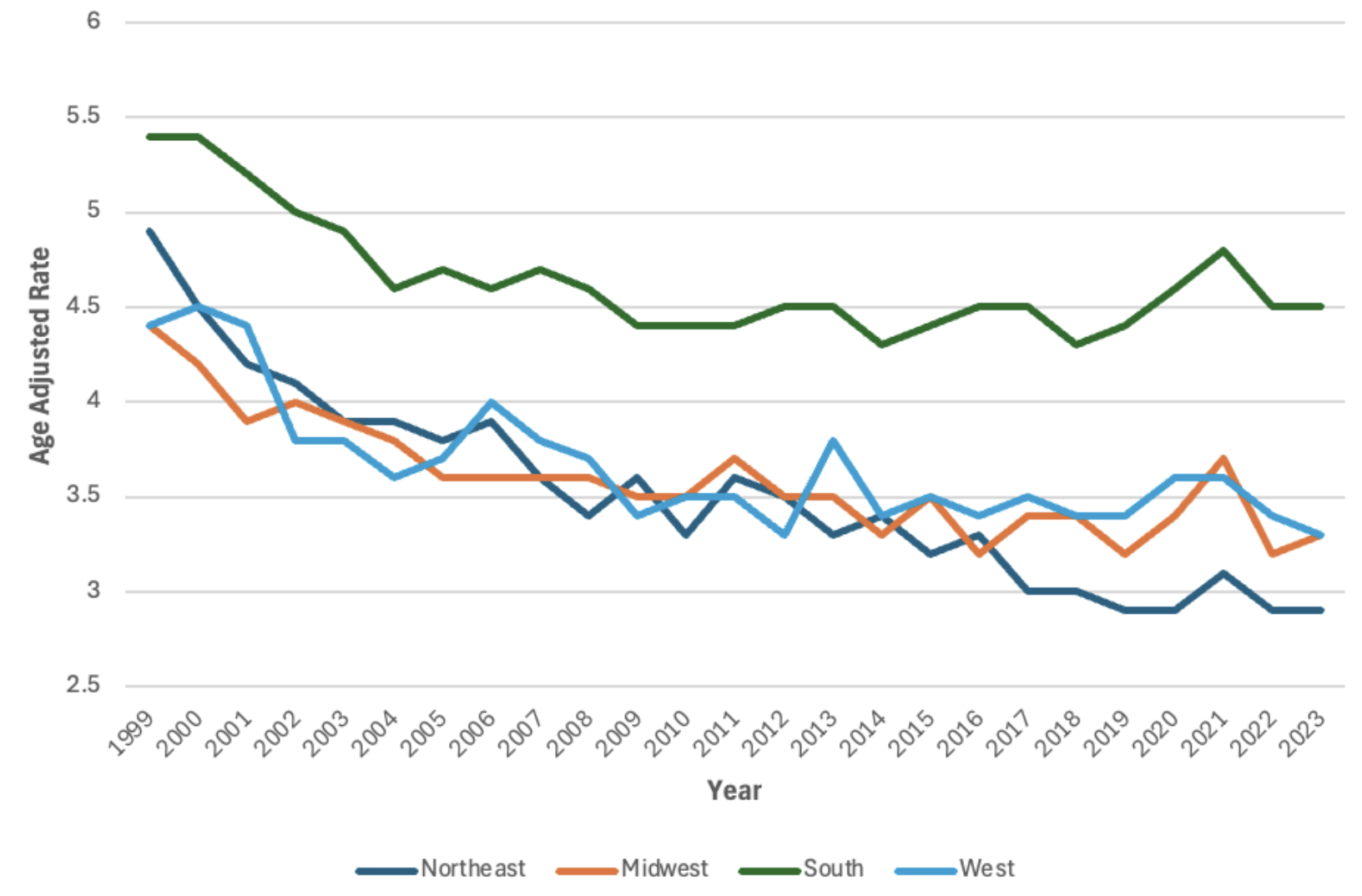

Supplement: Supplementary file 6 [file Image5.png]
